# Impact of early postoperative oral nutritional supplement utilization on clinical outcomes in colorectal surgery

**DOI:** 10.1186/s13741-020-00160-6

**Published:** 2020-10-05

**Authors:** David G. A. Williams, Tetsu Ohnuma, Vijay Krishnamoorthy, Karthik Raghunathan, Suela Sulo, Bridget A. Cassady, Refaat Hegazi, Paul E. Wischmeyer

**Affiliations:** 1 CAPER Unit, Department of Anesthesiology, Duke University School of Medicine, DUMC, Box 3094 Mail # 41, 2301 Erwin Road, 5692 HAFS, Durham, NC 27710 USA; 2 Duke Clinical Research Institute, Durham, NC USA; 3 Abbott Nutrition, Columbus, OH USA

**Keywords:** Health outcomes, Nutrition, Surgery, Oral nutrition supplement, Malnutrition, Postoperative, Colorectal surgery, Infection, Pneumonia, ICU

## Abstract

**Background:**

Small randomized trials of early postoperative oral nutritional supplementation (ONS) suggest various health benefits following colorectal surgery (CRS). However, real-world evidence of the impact of early ONS on clinical outcomes in CRS is lacking.

**Methods:**

Using a nationwide administrative-financial database (Premier Healthcare Database), we examined the association between early ONS use and postoperative clinical outcomes in patients undergoing elective open or laparoscopic CRS between 2008 and 2014. Early ONS was defined as the presence of charges for ONS before postoperative day (POD) 3. The primary outcome was composite infectious complications. Key secondary efficacy (intensive care unit (ICU) admission and gastrointestinal complications) and falsification (blood transfusion and myocardial infarction) outcomes were also examined. Propensity score matching was used to assemble patient groups that were comparable at baseline, and differences in outcomes were examined.

**Results:**

Overall, patients receiving early ONS were older with greater comorbidities and more likely to be Medicare beneficiaries with malnutrition. In a well-matched sample of early ONS recipients (*n* = 267) versus non-recipients (*n* = 534), infectious complications were significantly lower in early ONS recipients (6.7% vs. 11.8%, *P* < 0.03). Early ONS use was also associated with significantly reduced rates of pneumonia (*P* < 0.04), ICU admissions (*P* < 0.04), and gastrointestinal complications (*P* < 0.05). There were no significant differences in falsification outcomes.

**Conclusions:**

Although early postoperative ONS after CRS was more likely to be utilized in elderly patients with greater comorbidities, the use of early ONS was associated with reduced infectious complications, pneumonia, ICU admission, and gastrointestinal complications. This propensity score-matched study using real-world data suggests that clinical outcomes are improved with early ONS use, a simple and inexpensive intervention in CRS patients.

## Introduction

Perioperative malnutrition is a widely prevalent and potentially modifiable risk factor in patients undergoing colorectal surgery (CRS). Recent data indicate as many as 2 out of 3 patients are malnourished at the time of presentation for major gastrointestinal surgery, including CRS (Bruns et al. 2018; Wischmeyer et al. 2018). Further, perioperative malnutrition is a clinical predictor of postoperative mortality and morbidity in CRS. Malnutrition has been associated with increased hospital length of stay (LOS) (Garth et al. 2010), readmissions, costs of care (Bliss et al. 2015), and especially increased risk of postoperative infection (Bohl et al. 2016; Fukuda et al. 2015). Postoperative infection remains among the major complications following CRS (Smith et al. 2004), and quality improvement initiatives aimed at reducing surgical infections focus on appropriate administration of prophylactic antibiotics, perioperative hair clipping, normothermia (Arriaga et al. 2009; Berenguer et al. 2010), and early perioperative nutritional support (Wischmeyer et al. 2018).

Postoperative nutritional support is vital in maintaining nutritional status during the catabolic postoperative period and underscored by evidence for early oral intake following surgery as a routine part of ERAS protocols (Vlug et al. 2012; Weimann et al. 2006; Wischmeyer 2016). In fact, early oral intake has been identified as a key independent factor of improved outcomes following CRS (Vlug et al. 2012). Oral feeding is the preferred mode of nutrition for post-surgical patients (Weimann et al. 2006). Recovering postoperative patients, especially older adults, are challenged by decreased appetites, persistent nausea, opioid-induced constipation, and lack of education about how to optimize their diet (Wischmeyer 2016). Thus, nutritional therapy, often via oral nutritional supplements (ONS), may be required during the postoperative period following major surgery to avoid significant risk for the occurrence of postoperative malnutrition (Wischmeyer et al. 2018; Weimann et al. 2017). To address this, recent guidelines suggest that ONS should be routinely included in the postoperative care of gastrointestinal (GI) surgery patients to meet their nutritional needs (Wischmeyer et al. 2018). However, research evidence on clinical outcome data to support this recommendation is currently limited (Herbert et al. 2019).

ONS use in general hospitalized populations is variable between hospitals, and its use early during the postoperative period is currently not the routine standard of care. Prior small randomized controlled trials and older meta-analysis of such trials show initial benefits of early oral feeding (on postoperative day (POD) 1) (Herbert et al. 2019) versus delayed feeding, on outcomes for patients undergoing GI surgery. The more recent Cochrane review of early postoperative nutrition in lower GI surgery continues to indicate promise of early nutrition delivery for improved clinical outcomes, but indicates additional data is urgently needed (Herbert et al. 2019). Additional meta-analysis data has examined the specific use of perioperative immunonutrition (arginine-containing ONS). These data have consistently shown the benefit of perioperative immunonutrition primarily on infectious outcomes (Drover et al. 2011). Further, recent surgical nutrition guidelines emphasize the key role of high-protein delivery to improve recovery post-major abdominal surgery, such as CRS (Wischmeyer et al. 2018). Despite this, quite limited data exists from only few trials of solely high-protein ONS (non-immunonutrition) and this data show promise for improved outcomes from early postoperative high-protein ONS use (Keele et al. 1997; Yeung et al. 2017). However, large-scale real-world evidence is lacking to support the hypothesis that early postoperative ONS is associated with improved outcomes and is urgently needed to help support or refute the existing guideline recommendations (Wischmeyer et al. 2018). Thus, we examined the impact of early ONS (within 3 days of surgery) on postoperative infection and other clinical outcomes for patients undergoing CRS using a large administrative US healthcare database.

## Materials and methods

### Data source

The Premier Healthcare Database (PHD) contains cumulative data from over 970 contributing teaching and nonteaching hospitals/healthcare system hospitals across the USA (see https://products.premierinc.com/downloads/PremierHealthcareDatabaseWhitepaper.pdf for details). These hospitals vary in bed capacity and provide care to a largely urban population from all four geographic regions and their respective divisions as defined by the US Census. The PHD contains data on patient demographics, payer status, International Classification of Diseases (ICD) Diagnosis Codes, day-stamped Current Procedural Terminology (CPT) codes, and day-stamped hospital charge codes for every patient encounter for over 208 million unique patients.

### Population and study design

This was a retrospective cohort study among patients aged ≥ 18 years who underwent elective open or laparoscopic CRS between October 2008 and September 2014. Exclusion criteria included nonsurgical and outpatient surgical encounters (i.e., ambulatory surgeries and encounters with hospital LOS < 1 day); non-elective, non-colorectal surgeries; and encounters with missing ONS charges, or patients who experienced in-hospital death or required mechanical ventilation within the first three POD. This created an eligible study population of 61,031 patient encounters from 172 hospitals. The final study cohort was divided into groups of patients exposed and not exposed to early ONS following CRS.

### Exposures, outcomes, and covariates

Exposure to early ONS within the PHD was analyzed for inpatient encounters following CRS to determine the association between receiving ONS early during the postoperative period and subsequent clinical outcomes. The study exposure was early ONS, defined as receipt by POD 3 following colorectal surgery. To ensure that each encounter in the study cohort had the potential for exposure to ONS early during the postoperative period, we used tight exclusion criteria as previously described. Because there are no specific ICD-9/10 or CPT codes identifying ONS use, we relied on the PHD definition of “complete nutritional supplement, oral.” Product information under this definition was manually checked for accuracy. Enteral nutrition or tube feeding products were excluded, as were modular nutrient supplements (i.e., sole protein alone supplements or glutamine alone). The primary outcome was a composite of infectious complications, which was identified using ICD-9 codes (see Supplement 1 for full infectious outcome details). Secondary efficacy outcomes included ICU admission and GI complications as well as falsification outcomes such as red blood cell (RBC) transfusion and myocardial infarction. The falsification outcomes were assessed to examine non-biologically plausible outcomes that are highly unlikely to be related to an early postoperative ONS (Pizer 2016). A confirmed falsification test—in this case, an association between early ONS use and risks of these conditions—would suggest that perhaps residual confounding exists. Covariates considered in our analyses included malnutrition diagnosis, age, gender, race/ethnicity, insurance type (i.e., Medicare, Medicaid, and Managed Care Organizations (MCO), and others), hospital teaching status, hospital bed size, cancer, chronic obstructive pulmonary disease (COPD), chronic renal failure, vasopressor use, the van Walraven (VW) score, and data year. Comorbidity scores are validated and utilized in healthcare to classify a patient’s disease burden and predict mortality based on the type and number of patient comorbidities present (Thompson et al. 2015; Charlson et al. 1987). The VW score condenses the Elixhauser comorbidity system to a single numeric score that summarizes disease burden and is adequately discriminative for death in the hospital (van Walraven et al. 2009).

### Statistical analysis

Patients exposed to early ONS were identified and matched with greedy propensity score techniques (in a 1:2 fashion) (Austin 2014), to match exposed patients to unexposed patients to early ONS. A propensity score was built with the following variables: malnutrition, sex, age, VW score, payor category, race/ethnicity, cancer, renal failure, COPD, elective surgery, open surgery, vasopressor, hospital bed size, teaching hospital, and rural hospital. After matching, the standardized mean difference (SMD) was used to test the balance of covariates. A univariable logistic regression for binary outcomes was used to determine the association between early ONS exposure and clinical outcomes in the fully matched cohort. All analyses were performed via SAS version 9.4 (SAS Institute), and a *P* value of < 0.05 was considered statistically significant.

## Results

**Fig. 1:**
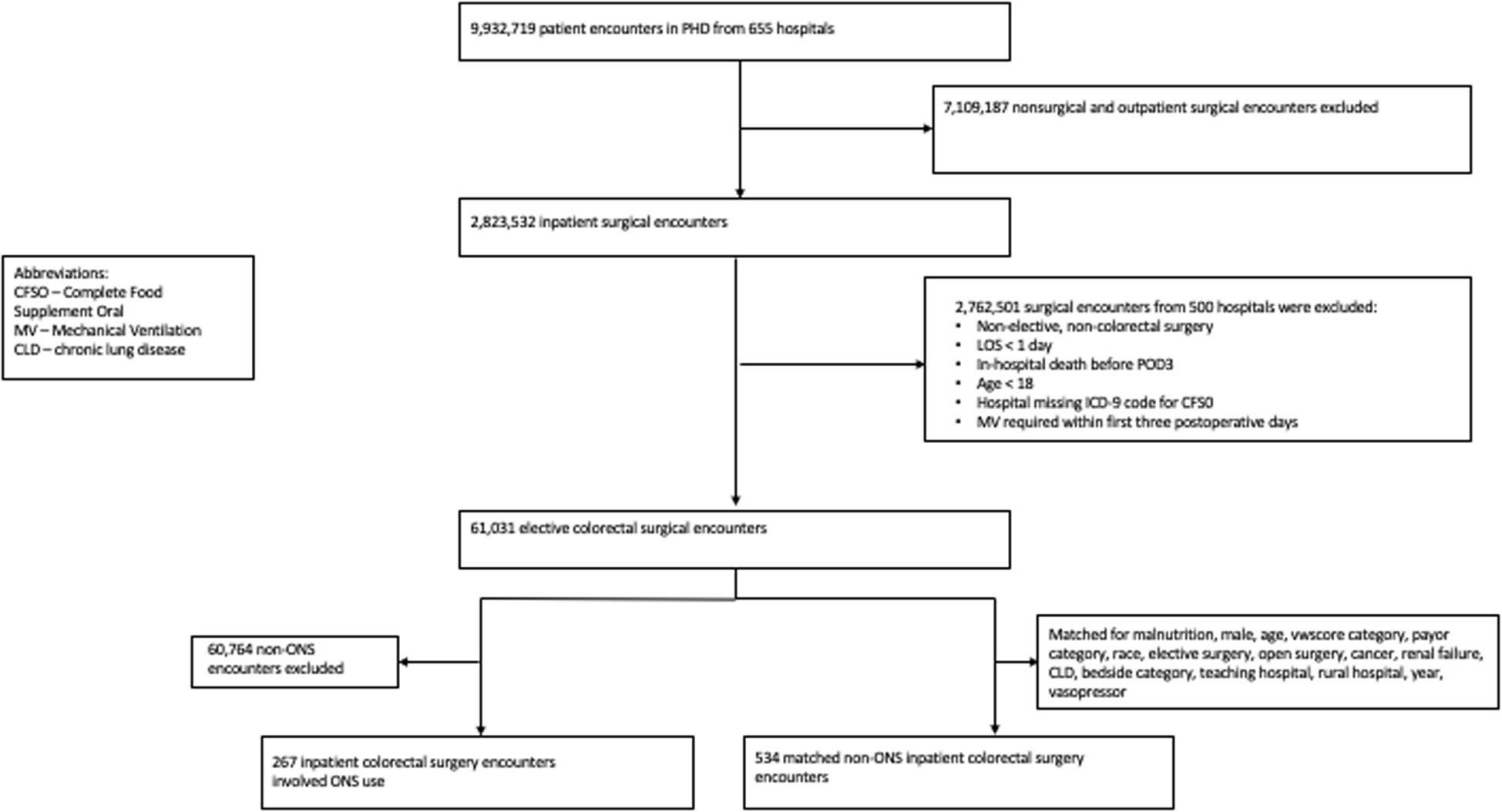
Patient flow and availability chart

Beginning with all patient encounters from 655 participating hospitals, the applied exclusion criteria resulted in 61,031 eligible patients (Fig. 1). ONS was provided in 267 (0.4%) of these patients. Of all patients eligible for ONS by POD 3, 5% had the diagnosis of malnutrition, 60% were aged ≥ 60 years, 48% were Medicare beneficiaries, 74% were Caucasian, 43% were from teaching hospitals, and 53% received care at mid-sized hospitals (Table 1). Outcome analysis in the overall cohort revealed 4% of patients required ICU admission after POD 3, 15% required RBC transfusion, 5% experienced infectious complications, 2% experienced pneumonia, 15% experienced GI complications, and the median hospital cost was $24,954 (Table 2).

**Table 1:** Baseline characteristics of colorectal surgery patients receiving oral nutritional supplement prior to matching

Characteristics	Before matching	
	Overall cohort (*n* = 61,031)	Column percentage
Malnutrition	3103	5%
Male	28,047	46%
Age group		
< 30	1142	2%
30–39	2783	5%
40–49	6734	11%
50–59	14,042	23%
60–69	16,507	27%
70–79	13,248	22%
≥80	6575	11%
VW score category		
< − 5	897	1%
− 5 to − 1	7563	12%
− 1 to 1	29,018	48%
1 to 5	9133	15%
> 5	14,420	24%
Comorbidities		
Cancer	12,333	20%
Renal failure	2444	4%
Chronic pulmonary disease	8440	14%
Payor category		
Managed care organization	21,682	36%
Medicaid	2353	4%
Medicare	29,018	48%
Others	7978	13%
Race		
Black	4953	8%
Hispanic	1030	2%
Others	9808	16%
White	45,240	74%
Open colorectal surgery	36,371	60%
Hospital bed size		
< 200	5446	9%
200–499	32,410	53%
≥ 500	23,175	38%
Teaching hospital	26,037	43%
Rural hospital	5644	9%
Vasopressor	17,381	28%

**Table 2:** Outcomes of colorectal surgery patient cohort receiving oral nutritional supplement prior to matching

Outcome	Before matching	
	Overall cohort (*n* = 61,031)	Column percentage
ICU admission after POD 3	2670	4%
In-hospital mortality	287	0%
Myocardial infarction	872	1%
RBC transfusion	9373	15%
Thrombosis (DVT, PE)	29	0%
Pneumonia	1501	2%
Infection	3133	5%
GI complication	9389	15%
30-day readmission	6797	11%
90-day readmission	10,123	17%
	Median	IQR
LOS	4	(3, 6)
Hospital cost	$24,953.88	($7472.57, $20,422.25)

*ICU* intensive care unit, *POD* postoperative day, *RBC* red blood cell, *DVT* deep vein thrombosis, *PE* pulmonary embolism, *GI* gastrointestinal, *LOS* length of stay, *IQR* interquartile range

### Propensity score matching

**Fig. 2:**
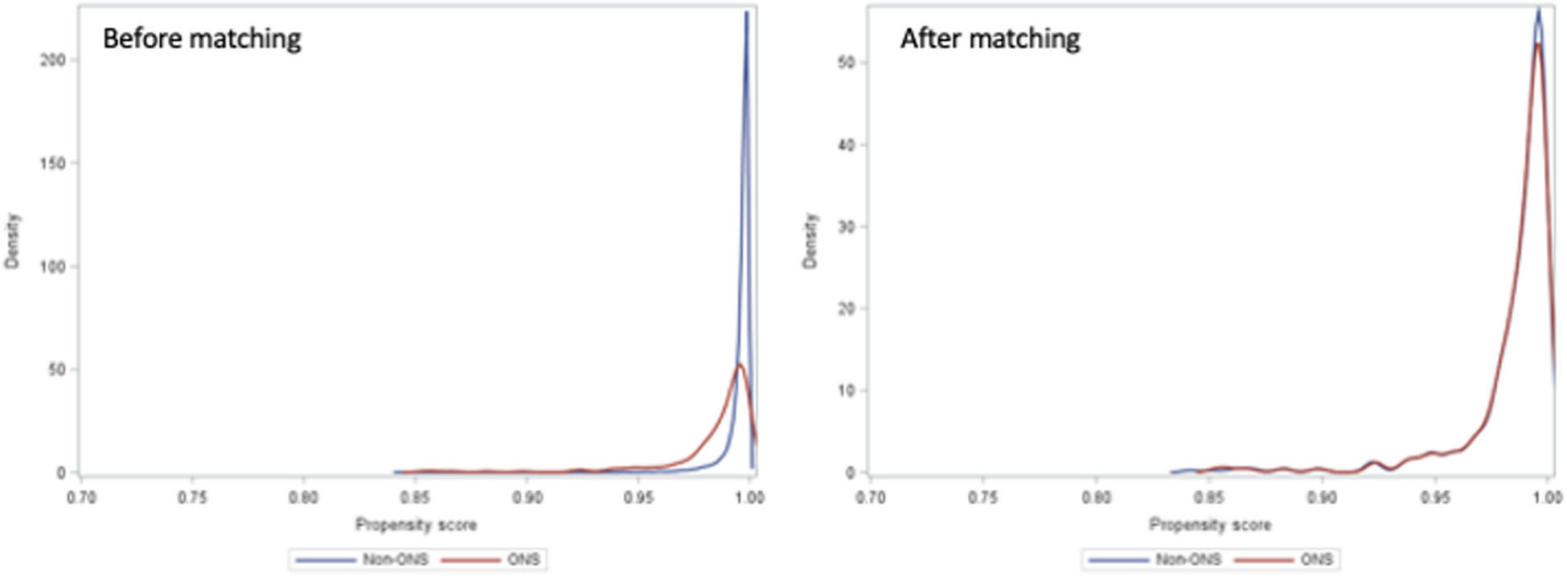
Kernel density plot with comparison of the non-ONS group (blue line) and the ONS group (red line) before (left panel) and after (right panel) propensity score matching

Propensity score matching produced a sample of 801 hospitalizations that were more balanced than the pre-matched sample in terms of observed characteristics such as age, sex, race/ethnicity, and comorbidities (Table 3). After matching, the two groups were comparable in terms of baseline characteristics (− 0.1 < SMD < 0.1) (Fig. 2).

**Table 3:** Baseline characteristics of colorectal surgery patient cohorts analyzed for ONS and non-ONS use after matching

Characteristics	After Matching				
	ONS (*n* = 267)	Percentage	Non-ONS (*n* = 534)	Percentage	SMD
Malnutrition	57	21.3	109	20.4	0.02
Male	125	46.8	247	46.3	0.01
Age group					0.13
< 30	11	4.1	21	3.9	
30–39	10	3.7	15	2.8	
40–49	10	3.7	18	3.4	
50–59	50	18.7	90	16.9	
60–69	60	22.5	130	24.3	
70–79	67	25.1	125	23.4	
≥ 80	59	22.1	135	25.3	
VW score category					0.17
< − 5	3	1.1			
− 5 to − 1	32	12	57	10.7	
− 1 to 1	95	35.6	214	40.1	
1 to 5	35	13.1	58	10.9	
> 5	102	38.2	205	38.4	
Comorbidities					0.00
Cancer	71	26.6	142	26.6	0.02
Renal failure	14	5.2	26	4.9	0.11
Chronic pulmonary disease	46	17.2	71	13.3	
Payor category					0.11
Managed care organization	61	22.8	114	21.3	
Medicaid	15	5.6	23	4.3	
Medicare	169	63.3	351	65.7	
Others	22	8.2	46	8.6	
Race					0.08
Black	25	9.4	38	7.1	
Hispanic	2	0.7	3	0.6	
Others	21	7.9	46	8.6	
White	219	82	447	83.7	
Open colorectal surgery	193	72.3	402	75.3	0.11
Hospital bed size					0.02
< 200	76	28.5	157	29.4	
200–499	132	49.4	255	47.8	
≥ 500	59	22.1	122	22.8	
Teaching hospital	69	25.8	138	25.8	0.00
Rural hospital	50	18.7	99	18.5	0.00
Vasopressor	108	40.4	216	40.4	0.00

### Association between early ONS exposure and reduced infectious complications

Early ONS exposure was associated with significantly fewer infectious complications (6.7% vs 11.7%, *P* < 0.03) and pneumonia rates (2.6% vs 6.2%, *P* < 0.04) in the matched sample (Table 4). Early ONS use was also associated with fewer ICU admissions (6% vs. 10%, *P* < 0.04) and fewer GI complications (16.5% vs 22.5%, *P* < 0.05). The matched sample did not, however, show a statistically significant difference in LOS and hospital costs associated with early ONS use.

**Table 4:** Outcomes of colorectal surgery patient cohorts analyzed for ONS and non-ONS use after matching

Outcome	After matching				
	ONS (*n* = 267)	Percentage	Non-ONS (*n* = 534)	Percentage	*P* value*
ICU after POD3	16	6	56	10.5	0.0384
In-hospital mortality	3	1.1	14	2.6	0.18
Myocardial infarction	5	1.9	18	3.4	0.24
RBC transfusion	87	32.6	147	27.5	0.14
Thrombosis (DVT, PE)	0	0	0	0	None
Pneumonia	7	2.6	33	6.2	0.034
Infection	18	6.7	63	11.8	0.027
GI complication	44	16.5	120	22.5	0.049
30-day readmission	34	12.7	68	12.7	1
90-day readmission	52	19.5	97	18.2	0.65
	Median	IQR	Median	IQR	
LOS	7	(4, 10)	6	(4, 9)	0.3471
Hospital cost	16,132.94	(11,472.1, 22,448.69)	14,279.46	(10,102.68, 19,844.54)	0.3454

*ICU* intensive care unit, *POD* postoperative day, *RBC* red blood cell, *DVT* deep vein thrombosis, *PE* pulmonary embolism, *GI* gastrointestinal, *LOS* length of stay, *IQR* interquartile range

*Univariable logistic regression for binary outcomes or paired *t* test for continuous outcomes

### Falsification variable analysis

Falsification variable analysis was used as described previously to assess for residual confounding in health outcome research (Prasad and Jena 2013). As described in a recent *JAMA* publication, pre-specified falsification end points, when confirmed, assist in validating true observational associations (Prasad and Jena 2013). We believe that it is highly unlikely for red blood cell transfusions and myocardial infarction to be causally related to early ONS use (i.e., we do not see an obvious causal pathway between early ONS use and these outcomes). Finding an association therefore would suggest possible residual confounding. We did not observe a statistically significant difference in RBC transfusion and myocardial infarction in the matched sample. This assists in supporting the validity of the biologically plausible finding that ONS was associated with decreased infectious complications.

## Discussion

Our real-world practice data shows, despite surgical guidelines encouraging early ONS utilization, ONS remains highly underutilized following CRS. Early ONS use was more frequently utilized in older CRS surgery patients with higher numbers of comorbid conditions. The disease acuity of patients receiving early ONS is further highlighted by the finding that 72% required open CRS. Yet, we observed a benefit in postoperative health outcomes from early ONS use, despite its more frequent use among patients with higher perioperative risk. Our key finding was exposure to early ONS was associated with reduced infectious complications including pneumonia. Further, early ONS utilization led to fewer ICU admissions and reduced GI complications. These real-world findings support the results from existing limited randomized controlled trial data in the postoperative CRS patients (Drover et al. 2011; Keele et al. 1997; Yeung et al. 2017).

Surgery exerts a significant catabolic stress characterized by the presence of an inflammatory response associated with mobilization of muscle amino acid stores and potentially conditionally essential nutrients, which is associated with a dysregulated immune response that increases the risk for postoperative complications, especially infectious complications (Hegazi et al. 2014). Both innate and adaptive immunity undergo change in response to the physiologic stress of surgery (Maung and Davis 2012). The severity of immune dysfunction is proportional to the extent of surgical trauma and depends on a number of factors, including the underlying disease requiring surgical treatment (e.g., cancer), coexisting infections, and impaired nutritional status. It is generally believed that major surgery is accompanied by sustained postoperative immunosuppression, which potentially increases the risk for infectious complications particularly in patients undergoing surgery for cancer (Dąbrowska and Słotwiński 2014). These data suggest that early ONS, administered by POD 3, may modulate the immune response to surgery and lead to reduced infectious complications.

These data provide key support to previous studies showing a signal of reduced infection as a result of the use of postoperative ONS, primarily immunonutrition (IMN) (Drover et al. 2011). A few recent studies demonstrated that IMN continues to show benefit, even in the context of modern enhanced recovery after surgery (ERAS) pathways. Moya et al. demonstrated a reduction in infectious complications (23.8% vs 10.7%; *P* = 0.0007), especially wound infections (16.4% vs 5.7%; *P* = 0.0008) with the use of IMN ONS when compared to standard high-calorie ONS (Moya et al. 2016). This trial delivered IMN ONS both pre- and postoperatively, whereas our data demonstrate an outcome benefit of postoperative ONS delivery alone on infectious complications. Our data reports the composite effect of all types of early ONS therapy and does not distinguish between IMN and standard ONS (including high-protein ONS). As previously described, very limited data exists for standard and high-protein ONS in CRS surgery. To our knowledge, only one recent trial was conducted examining postoperative standard ONS in CRS and demonstrated that postoperative high-protein ONS reduced LOS (Yeung et al. 2017). These data are among the first to demonstrate early ONS use, regardless of the specific formulation, improved infectious outcomes compared to patients not receiving ONS.

Early postoperative feeding in CRS has traditionally been challenged by perceived concerns for dysmotility, ileus, and postoperative gastrointestinal dysfunction (Wischmeyer et al. 2018; Petrini 2005; Artinyan et al. 2008). Traditionally, postoperative oral intake is delayed until clinical signs of the return of bowel function are present. Current ERAS practice, emphasizing immediate postoperative initiation of oral nutrition, challenges this dogma (Ljungqvist et al. 2017). Early oral feeding after CRS has been shown to be a key factor in improving bowel motility after surgery and reduces the incidence of postoperative paralytic ileus (Ng and Neill 2006). Finally, early oral intake (on POD 1) after GI surgery has been shown in a meta-analysis of multiple studies to significantly reduce mortality after GI surgery (when compared with patients who receive delayed feeding). In fact, surgical oncology patients who receive care with at least 70% of recommended ERAS pathways (including feeding on POD 1) have 42% improved 5-year survival versus patients not receiving ERAS care (Gustafsson et al. 2016). Early postoperative feeding along with appropriate fluid management was shown to be the key ERAS components essential to improving postoperative outcomes in this study. The potential confounding effect of the components of ERAS on the association of early ONS and postoperative outcomes is a valid research question that warrants further studies. Nonetheless, the current scientific literature supports that oral intake should be resumed as soon as possible after CRS.

The reduction in overall infection rates observed in this study potentially bears a financial consequence for hospitals as healthcare reimbursement progresses to a fee-for-value-based system (Aronson et al. 2018). The Centers for Medicare and Medicaid Services (CMS) linked payment to specific quality measures through both the Hospital-Acquired Condition (HAC) Reduction Program and the Hospital Value-Based Purchasing (VBP) Program (VanLare and Conway 2012; Calderwood et al. 2017). These two programs put hospitals at risk of losing over $1.9 billion in annual revenue through a reduction in payment to those deemed to be providing lower quality of care and a redistribution of payment to others deemed to be providing higher quality care. Surgical site infection (SSI) rates are one of the major hospital quality metrics tracked by these programs, which is key as SSI following CRS remains a significant challenge (Smith et al. 2004). Our data implies early ONS use following CRS as a feasible, practical clinical measure to improve this surgical outcome and provide financial savings to hospitals through cost avoidance.

This study has several limitations. First, PHD does not contain compliance data so it was not possible to discern how much of the prescribed ONS was consumed by patients. Next, it was also not possible to address whether certain ONS formulations were more advantageous than others since we relied on the PHD definition of “complete nutritional supplement, oral.” Also, while PHD is a representation of the broad surgical patient population and hospital systems that provide perioperative care, its generalizability may be limited. Propensity score methods can help address confounding by observable characteristics through balancing the exposure and treatment groups according to those observable characteristics. However, unobservable characteristics, which may be associated with the propensity to receive ONS and influence clinical outcomes (i.e., unmeasured health status and socioeconomic status) are not addressed by propensity score methods. Moreover, we cannot account for the percentage of patients receiving perioperative care as part of an ERAS pathway because there are no ICD or CPT codes that specify the ERAS program in its entirety, making it challenging to study in large administrative databases. Lastly, as this study is a retrospective cohort study, it allows only for causal inferences that are hypothesis generating for further prospective investigation.

## Conclusion

This large-scale study of real-world practice demonstrates benefits of providing early postoperative ONS in CRS patients. Our data show early postoperative ONS was more likely to be utilized in elderly patients, with greater comorbidities following CRS. Our key finding is that infectious complications were significantly reduced when early ONS was delivered in a well-matched sample of CRS patients. In addition, rates of pneumonia, ICU admission, and GI complications were decreased with early ONS use. Despite the observed benefits in this study, and surgical guidelines suggesting early postoperative ONS use in abdominal surgery, we observed limited ONS use in this high-risk group of surgical patients. These data suggest improved clinical outcomes and potential healthcare cost savings can be achieved from this simple and inexpensive clinical intervention. These findings also provide key initial data for future surgical nutrition clinical outcomes work and much needed randomized clinical trials and quality improvement studies in postoperative ONS use to improve patient health and economic outcomes.

## Supplementary information

**Additional file 1: Supplementary Table 1.** Infectious Complication ICD-9 codes.

## Data Availability

The data that support the findings of this study are available from Premier Health Inc but restrictions apply to the availability of these data, which were used under license for the current study, and so are not publicly available. Data are however available from the authors upon reasonable request and with permission of Premier Health Inc.
