# Supplementary material for: Impact of early postoperative oral nutritional supplement utilization on clinical outcomes in colorectal surgery
Source: Perioper Med (Lond). 2020 Oct 5;9:29. doi: 10.1186/s13741-020-00160-6 (PMC7534158; doi:10.1186/s13741-020-00160-6)
Supplement: Supplementary file 1 — Additional file 1: Supplementary Table 1. Infectious Complication ICD-9 codes. [file 13741_2020_160_MOESM1_ESM.docx]

**Supplementary Table 1.** Infectious Complication ICD-9 codes

| **ICD Code** | **ICD Description** | **ICD Major Category** | **ICD Major category Description** |
| --- | --- | --- | --- |
| 38 | SEPTICEMIA* | 11 | INFECTIOUS AND PARASITIC DISEASES |
| 38 | SEPTICEMIA, STREPTOCOCCAL | 11 | INFECTIOUS AND PARASITIC DISEASES |
| 38.1 | STAPHYLOCOCC SEPTICEMIA* | 11 | INFECTIOUS AND PARASITIC DISEASES |
| 38.1 | SEPTICEMIA, STAPHYLOCOCCAL NOS | 11 | INFECTIOUS AND PARASITIC DISEASES |
| 38.11 | SEPTICEMIA, MSSA STAPH AUREUS | 11 | INFECTIOUS AND PARASITIC DISEASES |
| 38.12 | SEPTICEMIA, MRSA RESIS STAPH AUREUS | 11 | INFECTIOUS AND PARASITIC DISEASES |
| 38.19 | SEPTICEMIA, STAPHYLOCOCCAL NEC | 11 | INFECTIOUS AND PARASITIC DISEASES |
| 38.2 | SEPTICEMIA, PNEUMOCOCCAL | 11 | INFECTIOUS AND PARASITIC DISEASES |
| 38.3 | SEPTICEMIA, ANAEROBIC | 11 | INFECTIOUS AND PARASITIC DISEASES |
| 38.4 | GRAM-NEG SEPTICEMIA NEC* | 11 | INFECTIOUS AND PARASITIC DISEASES |
| 38.4 | SEPTICEMIA, GRAM-NEG ORGANISM NOS | 11 | INFECTIOUS AND PARASITIC DISEASES |
| 38.41 | SEPTICEMIA, HEMOPHILUS INFLUENZAE | 11 | INFECTIOUS AND PARASITIC DISEASES |
| 38.42 | SEPTICEMIA, E. COLI | 11 | INFECTIOUS AND PARASITIC DISEASES |
| 38.43 | SEPTICEMIA, PSEUDOMONAS | 11 | INFECTIOUS AND PARASITIC DISEASES |
| 38.44 | SEPTICEMIA, SERRATIA | 11 | INFECTIOUS AND PARASITIC DISEASES |
| 38.49 | SEPTICEMIA, GRAM-NEG ORGANISM NEC | 11 | INFECTIOUS AND PARASITIC DISEASES |
| 38.8 | SEPTICEMIA NEC | 11 | INFECTIOUS AND PARASITIC DISEASES |
| 38.9 | SEPTICEMIA NOS | 11 | INFECTIOUS AND PARASITIC DISEASES |
| 41 | BACT INF IN OTH DIS/NOS* | 11 | INFECTIOUS AND PARASITIC DISEASES |
| 41 | STREPTOCOCCUS INFECT NOS | 11 | INFECTIOUS AND PARASITIC DISEASES |
| 41 | INFECTION, STREPTOCOCCUS NOS | 11 | INFECTIOUS AND PARASITIC DISEASES |
| 41.01 | INFECTION, STREPTOCOCCUS GROUP A | 11 | INFECTIOUS AND PARASITIC DISEASES |
| 41.02 | INFECTION, STREPTOCOCCUS GROUP B | 11 | INFECTIOUS AND PARASITIC DISEASES |
| 41.03 | INFECTION, STREPTOCOCCUS GROUP C | 11 | INFECTIOUS AND PARASITIC DISEASES |
| 41.04 | INFCT, STREP, GRP D [ENTEROCOCCUS] | 11 | INFECTIOUS AND PARASITIC DISEASES |
| 41.05 | INFECTION, STREPTOCOCCUS GROUP G | 11 | INFECTIOUS AND PARASITIC DISEASES |
| 41.09 | INFECTION, STREPTOCOCCUS NEC | 11 | INFECTIOUS AND PARASITIC DISEASES |
| 41.1 | STAPH INFECTION NOS* | 11 | INFECTIOUS AND PARASITIC DISEASES |
| 41.1 | INFECTION, STAPHYLOCOCCUS NOS | 11 | INFECTIOUS AND PARASITIC DISEASES |
| 41.11 | INFECTION, MSSA STAPH AUREUS | 11 | INFECTIOUS AND PARASITIC DISEASES |
| 41.12 | INFECTION, MRSA STAPH AUREUS | 11 | INFECTIOUS AND PARASITIC DISEASES |
| 41.19 | INFECTION, STAPHYLOCOCCUS NEC | 11 | INFECTIOUS AND PARASITIC DISEASES |
| 41.2 | INFECTION, PNEUMOCOCCUS | 11 | INFECTIOUS AND PARASITIC DISEASES |
| 41.3 | KLEBSIELLA PNEUMONIAE | 11 | INFECTIOUS AND PARASITIC DISEASES |
| 41.4 | INFECTION, E. COLI NOS | 11 | INFECTIOUS AND PARASITIC DISEASES |
| 41.41 | SHIGA TOXIN-PRODUCING E. COLI | 11 | INFECTIOUS AND PARASITIC DISEASES |
| 41.42 | OTHER SHIGA TOXIN-PRODUCING E. COLI | 11 | INFECTIOUS AND PARASITIC DISEASES |
| 41.43 | SHIGA TOXIN-PRODUCING E. COLI UNSPE | 11 | INFECTIOUS AND PARASITIC DISEASES |
| 41.49 | OTHER UNSPEC SHIGA TOXIN E. COLI | 11 | INFECTIOUS AND PARASITIC DISEASES |
| 41.5 | INFECTION, HEMOPHILUS INFLU | 11 | INFECTIOUS AND PARASITIC DISEASES |
| 41.6 | INFECTION, PROTEUS | 11 | INFECTIOUS AND PARASITIC DISEASES |
| 41.7 | INFECTION, PSEUDOMONAS | 11 | INFECTIOUS AND PARASITIC DISEASES |
| 41.8 | BACTERIAL INFECTION NEC* | 11 | INFECTIOUS AND PARASITIC DISEASES |
| 41.81 | INFECTION, BACTERIAL D/T MYCOPLASMA | 11 | INFECTIOUS AND PARASITIC DISEASES |
| 41.82 | INFCT BCTRL D/T BACTEROIDES FRAGILI | 11 | INFECTIOUS AND PARASITIC DISEASES |
| 41.83 | INFCT, BCTRL D/T C. PERFRINGENS | 11 | INFECTIOUS AND PARASITIC DISEASES |
| 41.84 | INFCT, BCTRL D/T OTHER ANAEROBES | 11 | INFECTIOUS AND PARASITIC DISEASES |
| 41.85 | INFCT, BCTRL D/T OTH G-NEG ORGNSM | 11 | INFECTIOUS AND PARASITIC DISEASES |
| 41.86 | INFCT BCTRL D/T HELICOBACTER PYLORI | 11 | INFECTIOUS AND PARASITIC DISEASES |
| 41.89 | INFECTION, BACTERIAL NEC | 11 | INFECTIOUS AND PARASITIC DISEASES |
| 41.9 | INFECTION, BACTERIAL NOS | 11 | INFECTIOUS AND PARASITIC DISEASES |
| 480 | VIRAL PNEUMONIA* | 24 | RESPIRATORY SYSTEM DISEASES |
| 480 | PNEUMONIA, ADENOVIRUS | 24 | RESPIRATORY SYSTEM DISEASES |
| 480.1 | PNEUMONIA D/T RSPRT SYNCYTIAL VIRUS | 24 | RESPIRATORY SYSTEM DISEASES |
| 480.2 | PNEUMONIA D/T PARAINFLUENZA VIRUS | 24 | RESPIRATORY SYSTEM DISEASES |
| 480.8 | PNEUMONIA D/T VIRUS NEC | 24 | RESPIRATORY SYSTEM DISEASES |
| 480.9 | PNEUMONIA D/T VIRUS NOS | 24 | RESPIRATORY SYSTEM DISEASES |
| 481 | PNEUMONIA D/T PNEUMOCOCCAL | 24 | RESPIRATORY SYSTEM DISEASES |
| 482 | OTH BACTERIAL PNEUMONIA* | 24 | RESPIRATORY SYSTEM DISEASES |
| 482 | PNEUMONIA D/T KLEBSIELLA PNEUMONIAE | 24 | RESPIRATORY SYSTEM DISEASES |
| 482.1 | PNEUMONIA D/T PSEUDOMONAS | 24 | RESPIRATORY SYSTEM DISEASES |
| 482.2 | PNEUMONIA D/T HEMOPHILUS INFLUENZAE | 24 | RESPIRATORY SYSTEM DISEASES |
| 482.3 | STREPTOCOCCAL PNEUMONIA* | 24 | RESPIRATORY SYSTEM DISEASES |
| 482.3 | PNEUMONIA D/T STREPTOCOCCUS NOS | 24 | RESPIRATORY SYSTEM DISEASES |
| 482.31 | PNEUMONIA D/T STREPTOCOCCUS GROUP A | 24 | RESPIRATORY SYSTEM DISEASES |
| 482.32 | PNEUMONIA D/T STREPTOCOCCUS GROUP B | 24 | RESPIRATORY SYSTEM DISEASES |
| 482.39 | PNEUMONIA D/T STREPTOCOCCUS NEC | 24 | RESPIRATORY SYSTEM DISEASES |
| 482.4 | STAPHYLOCOCCAL PNEUMONIA | 24 | RESPIRATORY SYSTEM DISEASES |
| 482.4 | PNEUNONIA D/T STAPHYLOCOCCUS NOS | 24 | RESPIRATORY SYSTEM DISEASES |
| 482.41 | METHCLLN SUSP PNEU D/T STAPH AUREUS | 24 | RESPIRATORY SYSTEM DISEASES |
| 482.42 | METHCLLN REST PNEU D/T STAPH AUREUS | 24 | RESPIRATORY SYSTEM DISEASES |
| 482.49 | PNEUMONIA D/T STAPHYLOCOCCUS NEC | 24 | RESPIRATORY SYSTEM DISEASES |
| 482.8 | BACTERIAL PNEUMONIA NEC* | 24 | RESPIRATORY SYSTEM DISEASES |
| 482.81 | PNEUMONIA D/T ANAEROBES | 24 | RESPIRATORY SYSTEM DISEASES |
| 482.82 | PNEUMONIA D/T ESCHERICHIA COLI | 24 | RESPIRATORY SYSTEM DISEASES |
| 482.83 | PNEUMONIA D/T GRAM-NEGATIVE NEC | 24 | RESPIRATORY SYSTEM DISEASES |
| 482.84 | PNEUMONIA D/T LEGIONNAIRES' DISEASE | 24 | RESPIRATORY SYSTEM DISEASES |
| 482.89 | PNEUMONIA, BACTERIAL NEC | 24 | RESPIRATORY SYSTEM DISEASES |
| 482.9 | PNEUMONIA, BACTERIAL NOS | 24 | RESPIRATORY SYSTEM DISEASES |
| 483 | PNEUMONIA: ORGANISM NEC* | 24 | RESPIRATORY SYSTEM DISEASES |
| 483 | PNEUMONIA D/T MYCOPLASMA PNEUMONIAE | 24 | RESPIRATORY SYSTEM DISEASES |
| 483.1 | PNEUMONIA D/T CHLAMYDIA | 24 | RESPIRATORY SYSTEM DISEASES |
| 483.8 | PNEUMONIA D/T ORGANISM NEC | 24 | RESPIRATORY SYSTEM DISEASES |
| 484 | PNEUM IN OTH INFEC DIS* | 24 | RESPIRATORY SYSTEM DISEASES |
| 484.1 | PNEUMONIA IN CYTOMEGALIC INCLS DIS | 24 | RESPIRATORY SYSTEM DISEASES |
| 484.3 | PNEUMONIA IN WHOOPING COUGH | 24 | RESPIRATORY SYSTEM DISEASES |
| 484.5 | PNEUMONIA IN ANTHRAX | 24 | RESPIRATORY SYSTEM DISEASES |
| 484.6 | PNEUMONIA IN ASPERGILLOSIS | 24 | RESPIRATORY SYSTEM DISEASES |
| 484.7 | PNEUMONIA IN SYSTEMIC MYCOSES | 24 | RESPIRATORY SYSTEM DISEASES |
| 484.8 | PNEUMONIA IN OTH INFCT DISEASE CE | 24 | RESPIRATORY SYSTEM DISEASES |
| 485 | BRONCHOPNEUMONIA, ORGANISM NOS | 24 | RESPIRATORY SYSTEM DISEASES |
| 486 | PNEUMONIA, ORGANISM NOS | 24 | RESPIRATORY SYSTEM DISEASES |
| 487 | INFLUENZA* | 24 | RESPIRATORY SYSTEM DISEASES |
| 487 | INFLUENZA W/PNEUMONIA | 24 | RESPIRATORY SYSTEM DISEASES |
| 487.1 | INFLUENZA W/RSPRT MNFST NEC | 24 | RESPIRATORY SYSTEM DISEASES |
| 487.8 | INFLUENZA W/MANIFESTATION NEC | 24 | RESPIRATORY SYSTEM DISEASES |
| 510 | EMPYEMA* | 24 | RESPIRATORY SYSTEM DISEASES |
| 510 | EMPYEMA W/FISTULA | 24 | RESPIRATORY SYSTEM DISEASES |
| 510.9 | EMPYEMA W/O FISTULA | 24 | RESPIRATORY SYSTEM DISEASES |
| 519.2 | MEDIASTINITIS | 24 | RESPIRATORY SYSTEM DISEASES |
| 567 | PERITONITIS IN INFECTIOUS DISEASE | 6 | DIGESTIVE SYSTEM DISEASES |
| 567.22 | PERITONEAL ABSCESS | 6 | DIGESTIVE SYSTEM DISEASES |
| 567.29 | PERITONITIS, SUPPURATIVE NEC | 6 | DIGESTIVE SYSTEM DISEASES |
| 569.5 | ABSCESS, INTESTINE | 6 | DIGESTIVE SYSTEM DISEASES |
| 590.1 | ACUTE PYELONEPHRITIS* | 10 | GENITOURINARY SYSTEM DISEASES |
| 590.2 | ABSCESS, RENAL/PERINEPHRIC | 10 | GENITOURINARY SYSTEM DISEASES |
| 590.8 | OTHER PYELONEPHRITIS* | 10 | GENITOURINARY SYSTEM DISEASES |
| 590.9 | INFECTION, KIDNEY NOS | 10 | GENITOURINARY SYSTEM DISEASES |
| 595 | CYSTITIS, ACUTE | 10 | GENITOURINARY SYSTEM DISEASES |
| 595.9 | CYSTITIS NOS | 10 | GENITOURINARY SYSTEM DISEASES |
| 599 | INFECTION, URINARY TRACT NOS | 10 | GENITOURINARY SYSTEM DISEASES |
| 785.5 | SYMPTOM, SHOCK NOS | 27 | SYMPTOMS, SIGNS, AND ILL-DEFINED CONDITIONS |
| 785.52 | SYMPTOM, SHOCK, SEPTIC | 27 | SYMPTOMS, SIGNS, AND ILL-DEFINED CONDITIONS |
| 785.59 | SYMPTOM, SHOCK W/O TRAUMA NEC | 27 | SYMPTOMS, SIGNS, AND ILL-DEFINED CONDITIONS |
| 790.7 | BACTEREMIA | 27 | SYMPTOMS, SIGNS, AND ILL-DEFINED CONDITIONS |
| 958.4 | SHOCK, TRAUMATIC | 17 | INJURY AND POISONING |
| 995.91 | ADVEF SIRS D/T SEPSIS | 17 | INJURY AND POISONING |
| 995.92 | ADVEF SIRS D/T SEVERE SEPSIS | 17 | INJURY AND POISONING |
| 996 | REPLACE & GRAFT COMPLIC* | 3 | COMPLICATIONS |
| 996 | MALFUNC CARD DEVIC/GRAFT | 3 | COMPLICATIONS |
| 996 | MALFUNCTION, CARDIAC DEV/GRAFT NOS | 17 | INJURY AND POISONING |
| 996.01 | MALFUNCTION, CARDIAC PACEMAKER | 17 | INJURY AND POISONING |
| 996.02 | MALFUNCTION, PROSTHETIC HEART VALVE | 17 | INJURY AND POISONING |
| 996.03 | MALFUNCTION, CORONARY BYPASS GRAFT | 17 | INJURY AND POISONING |
| 996.04 | COMPLICATIONS D/T AICD | 17 | INJURY AND POISONING |
| 996.09 | MALFUNCTION, CARDIAC DEV/GRAFT NEC | 17 | INJURY AND POISONING |
| 996.1 | MALFUNCTION, VASCULAR DEVICE/GRAFT | 17 | INJURY AND POISONING |
| 996.2 | MALFUNCTION NRV SYST DEVICE/GRAFT | 17 | INJURY AND POISONING |
| 996.3 | MALFUNC GU DEVICE/GRAFT* | 3 | COMPLICATIONS |
| 996.3 | MALFUNCTION GU DEVICE/GRAFT NOS | 17 | INJURY AND POISONING |
| 996.31 | MALFUNCTION URETHRAL CATHETER | 17 | INJURY AND POISONING |
| 996.32 | MALFUNCTION, IUD | 17 | INJURY AND POISONING |
| 996.39 | MALFUNCTION GU DEVICE/GRAFT NEC | 17 | INJURY AND POISONING |
| 996.4 | MECH COMP INTRL ORTHOPEDIC DEVICE IMPLAN | 3 | COMPLICATIONS |
| 996.4 | MECH CMPL INTRN ORTH DEV/GRAFT NOS | 17 | INJURY AND POISONING |
| 996.41 | MECH LOOSENING, PROSTHETIC JOINT | 17 | INJURY AND POISONING |
| 996.42 | DISLOCATION, PROSTHETIC JOINT | 17 | INJURY AND POISONING |
| 996.43 | BROKEN PROSTHETIC JOINT IMPLANT | 17 | INJURY AND POISONING |
| 996.44 | FX, PERI-PRSTH, AROUND PRSTH JOINT | 17 | INJURY AND POISONING |
| 996.45 | OSTEOLYSIS, PERI-PROSTHETIC | 17 | INJURY AND POISONING |
| 996.46 | WEAR PRSTH JNT ARTCLR BEARING SRFC | 17 | INJURY AND POISONING |
| 996.47 | MECH CMPL, PRSTH JOINT IMPLANT NEC | 17 | INJURY AND POISONING |
| 996.49 | MECH CMPL INTRN ORTH DEV/GRAFT NEC | 17 | INJURY AND POISONING |
| 996.5 | ME-COMP OTH SPEC PROS DEV IM/ | 3 | COMPLICATIONS |
| 996.51 | MALFUNCTION, CORNEAL GRAFT | 17 | INJURY AND POISONING |
| 996.52 | MALFUNCTION, OTHER TISSUE GRAFT | 17 | INJURY AND POISONING |
| 996.53 | MALFUNCTION, LENS PROSTHESIS | 17 | INJURY AND POISONING |
| 996.54 | MALFUNCTION, BREAST PROSTHESIS | 17 | INJURY AND POISONING |
| 996.55 | CMPL D/T ARTIFICIAL SKIN GRAFT | 17 | INJURY AND POISONING |
| 996.56 | CMPL D/T PERITONEAL DIALYSIS CATH | 17 | INJURY AND POISONING |
| 996.57 | COMPLICATIONS DUE TO INSULIN PUMP | 17 | INJURY AND POISONING |
| 996.59 | MALFUNCTION OTHER DEVICE/GRAFT | 17 | INJURY AND POISONING |
| 996.6 | INFEC/INFLAM-DEVIC/GRAFT | 3 | COMPLICATIONS |
| 996.6 | INFCT D/T UNSPECIFIED DEVICE/GRAFT | 17 | INJURY AND POISONING |
| 996.61 | INFCT D/T CARDIAC DEVICE/GRAFT | 17 | INJURY AND POISONING |
| 996.62 | INFCT D/T OTH VASCULAR DEV/GRAFT | 17 | INJURY AND POISONING |
| 996.63 | INFECTION D/T NERV SYST DEV/GRAFT | 17 | INJURY AND POISONING |
| 996.64 | INFCT D/T INDWELLING URINE CATHETER | 17 | INJURY AND POISONING |
| 996.65 | INFCT D/T OTH GENITOURINARY DEVICE | 17 | INJURY AND POISONING |
| 996.66 | INFCT D/T INTERNAL JOINT PROSTHESIS | 17 | INJURY AND POISONING |
| 996.67 | INFCT D/T INTERNAL ORTH DEVICE NEC | 17 | INJURY AND POISONING |
| 996.68 | INFET D/T PERITONEAL DIALYSIS CATH | 17 | INJURY AND POISONING |
| 996.69 | INFCT D/T INTRN PRSTH DEVICE NEC | 17 | INJURY AND POISONING |
| 996.7 | COMPLIC DEVICE/GRAFT NEC | 3 | COMPLICATIONS |
| 996.7 | CMPL NEC D/T UNSPC DEVICE/GRAFT | 17 | INJURY AND POISONING |
| 996.71 | COMPLICATION, HEART VALVE PROSTHES | 17 | INJURY AND POISONING |
| 996.72 | COMPLICATION, OTHER CARDIAC DEVICE | 17 | INJURY AND POISONING |
| 996.73 | COMPLICATION, RENAL DIALYS DEV/GRFT | 17 | INJURY AND POISONING |
| 996.74 | COMPLICATION, OTH VASCULAR DEV/GRFT | 17 | INJURY AND POISONING |
| 996.75 | COMPLICATION, NEURO DEVICE/GRAFT | 17 | INJURY AND POISONING |
| 996.76 | COMPLICATION, GU DEVICE/GRAFT | 17 | INJURY AND POISONING |
| 996.77 | COMPLICATION, INT JOINT PROSTHESIS | 17 | INJURY AND POISONING |
| 996.78 | COMPLICATION, OTH INT ORTH DEVICE | 17 | INJURY AND POISONING |
| 996.79 | COMPLICATION, INT PROSTH DEVICE NEC | 17 | INJURY AND POISONING |
| 996.8 | COMP TRANSP ORG | 3 | COMPLICATIONS |
| 996.8 | COMPLICATION, ORGAN TRANSPLANT NOS | 17 | INJURY AND POISONING |
| 996.81 | COMPLICATION, KIDNEY TRANSPLANT | 17 | INJURY AND POISONING |
| 996.82 | COMPLICATION LIVER TRANSPLANT | 17 | INJURY AND POISONING |
| 996.83 | COMPLICATION, HEART TRANSPLANT | 17 | INJURY AND POISONING |
| 996.84 | COMPLICATION, TRANSPLANTED LUNG | 17 | INJURY AND POISONING |
| 996.85 | COMPLICATION, BONE MARROW TRANSPLNT | 17 | INJURY AND POISONING |
| 996.86 | COMPLICATION, PANCREAS TRANSPLANT | 17 | INJURY AND POISONING |
| 996.87 | COMPLICATION, INTESTINE TRANSPLANT | 17 | INJURY AND POISONING |
| 996.88 | COMPLICATION, STEM CELL | 17 | INJURY AND POISONING |
| 996.89 | COMPLICATION, OTH ORGAN TRANSPLANT | 17 | INJURY AND POISONING |
| 996.9 | COMPL REATTACHED PART* | 3 | COMPLICATIONS |
| 996.9 | COMPLICATION, REATTACHED EXTREM NOS | 17 | INJURY AND POISONING |
| 996.91 | COMPLICATION, REATTACHED FOREARM | 17 | INJURY AND POISONING |
| 996.92 | COMPLICATION, REATTACHED HAND | 17 | INJURY AND POISONING |
| 996.93 | COMPLICATION, REATTACHED FINGER | 17 | INJURY AND POISONING |
| 996.94 | COMPLICATION, REATTACHED ARM NEC | 17 | INJURY AND POISONING |
| 996.95 | COMPLICATION, REATTACHED FOOT/TOE | 17 | INJURY AND POISONING |
| 996.96 | COMPLICATION, REATTACHED LEG NEC | 17 | INJURY AND POISONING |
| 996.99 | COMPLICATION, REATTACHED PART NEC | 17 | INJURY AND POISONING |
| 997.31 | VENTILATOR ASSOCIATED PNEUMONIA | 17 | INJURY AND POISONING |
| 998 | SHOCK, POSTOPERATIVE | 17 | INJURY AND POISONING |
| 998.3 | POSTOP WOUND DISRUPTION | 3 | COMPLICATIONS |
| 998.3 | DISRUPTION OF WOUND, UNSPECIFIED | 17 | INJURY AND POISONING |
| 998.31 | DISRUPT, INTERN OPER SURG WOUND | 17 | INJURY AND POISONING |
| 998.32 | DISRUPT, EXTERNAL OPER SURG WOUND | 17 | INJURY AND POISONING |
| 998.33 | DISRUPT TRAUMAT INJURY WOUND REPAIR | 17 | INJURY AND POISONING |
| 998.5 | POSTOPERATIVE INFECTION* | 3 | COMPLICATIONS |
| 998.89 | COMPLICATIONS, SURGICAL NEC | 17 | INJURY AND POISONING |
| 999.3 | OTHER INFECTION DUE TO MEDICAL CARE NEC | 17 | INJURY AND POISONING |
